# Aggregation Strategy on Federated Machine Learning Algorithm for Collaborative Predictive Maintenance

**DOI:** 10.3390/s22166252

**Published:** 2022-08-19

**Authors:** Ali Bemani, Niclas Björsell

**Affiliations:** Department of Electrical Engineering, Mathematics and Science, University of Gävle, 80176 Gävle, Sweden

**Keywords:** distributed machine learning algorithm, edge and fog computing, federated learning, resource allocation, aggregation strategy

## Abstract

Industry 4.0 lets the industry build compact, precise, and connected assets and also has made modern industrial assets a massive source of data that can be used in process optimization, defining product quality, and predictive maintenance (PM). Large amounts of data are collected from machines, processed, and analyzed by different machine learning (ML) algorithms to achieve effective PM. These machines, assumed as edge devices, transmit their data readings to the cloud for processing and modeling. Transmitting massive amounts of data between edge and cloud is costly, increases latency, and causes privacy concerns. To address this issue, efforts have been made to use edge computing in PM applications., reducing data transmission costs and increasing processing speed. Federated learning (FL) has been proposed a mechanism that provides the ability to create a model from distributed data in edge, fog, and cloud layers without violating privacy and offers new opportunities for a collaborative approach to PM applications. However, FL has challenges in confronting with asset management in the industry, especially in the PM applications, which need to be considered in order to be fully compatible with these applications. This study describes distributed ML for PM applications and proposes two federated algorithms: Federated support vector machine (FedSVM) with memory for anomaly detection and federated long-short term memory (FedLSTM) for remaining useful life (RUL) estimation that enables factories at the fog level to maximize their PM models’ accuracy without compromising their privacy. A global model at the cloud level has also been generated based on these algorithms. We have evaluated the approach using the Commercial Modular Aero-Propulsion System Simulation (CMAPSS) dataset to predict engines’ RUL Experimental results demonstrate the advantage of FedSVM and FedLSTM in terms of model accuracy, model convergence time, and network usage resources.

## 1. Introduction

In recent years, the number of Internet of Things (IoT) devices has increased dramatically due to the rapid advances in hardware, software, and wireless communications technology, which provides data observation and measurement from physical work to cyber work. An analysis in [1] shows that the IoT has a potential economic impact of between USD 3.9 trillion and USD 11.1 trillion per year by 2025. Ericsson predicts that by 2050, there will be 24 billion internet-connected devices around the world. It means almost every object around us will be connected through wireless communication. In the past, we have seen the process of transferring computing, data storage, and applications to cloud data centers. In this way, an application has access to shared computing and storage resources on demand. The cloud computing model has several advantages: shared resources, low cost in deploying, high scalability, accessibility, and availability. IoT devices usually have limited computing power and small memory, which are widely used in smart houses, smart cities, autonomous vehicle driving, industries, manufacturing, and industrial internet of things. IoT devices continuously generate large amounts of data that need to be collected and analyzed [2]. Transferring such a large amount of data to cloud servers increases communication cost and network bandwidth usage, causes delayed system response, and puts data privacy at risk. To solve this issue, fog or edge computing has been proposed, in which data analysis and computation happen at such places where there is close proximity to the physical location that data originate from [3].

**Figure 1:**
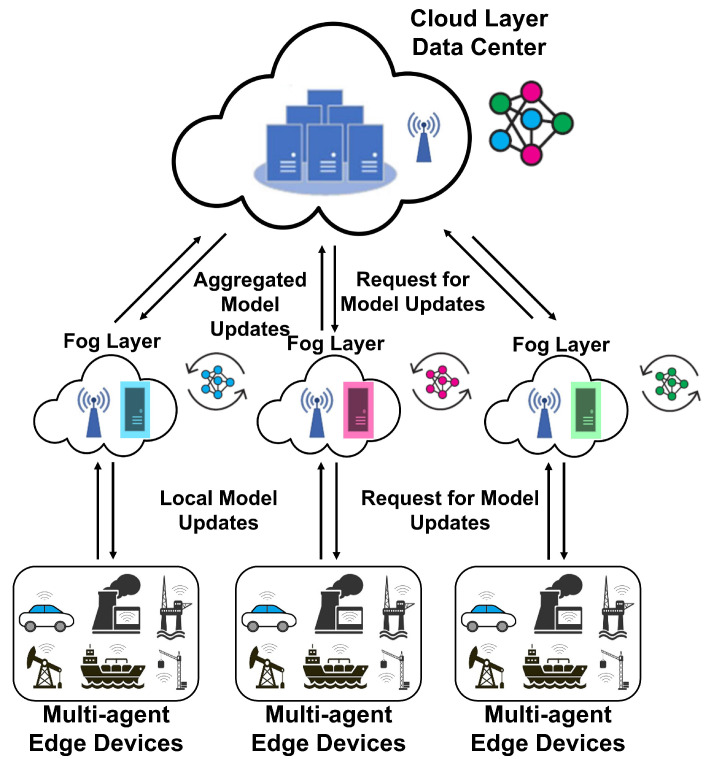
Hierarchical and collaborative edge-fog-cloud architecture.

The hierarchical and collaborative edge-fog-cloud architecture, which is shown in Figure 1, has a great benefit. It enables us to use a distributed machine learning (DML) algorithm to achieve an optimal solution while satisfying the given constraint over the network, e.g., limitation on bandwidth usage, communications cost, delay, and packet drops [4].

The digital transformation of production and manufacturing, industry 4.0 (I4.0), uses information and communication technology to create intelligent industries. I4.0 shares this vision that many infrastructure technologies underlying cyber-physical systems and data analytics are converged into a new distributed and automated dynamic network. The generated data from IoT devices facilitate information visibility and process automation in industrial digitalization. One of the applications that are primarily used in I4.0 is to predict the failure of manufacturing equipment. PM allows the business owner to decide whether to repair or replace the component before an actual breakdown affects the entire production line. Therefore, I4.0 requires effective asset management to optimize the distribution of tasks for PM models [5].

To achieve effective PM, huge amounts of data should be collected, processed, and ultimately analyzed by an ML algorithm. Edge and fog computing can process data with the use of distributed algorithms and provides opportunities to reduce data transfer costs and increase processing speed, especially in PM applications [6]. The authors in [7] present three main techniques that use distributed machine learning algorithms and data processing on intermediate nodes. These techniques are categorized according to the place where the data are processed: Edge, Fog, and Cloud.

Since edge computing has emerged as an essential paradigm for IoT-based systems, efforts are being made to use devices at the edge of the network to perform computations as much as possible, rather than using the cloud to process data only [8].

FL is a kind of collaborative ML without centralized training data which aims to train a global model from distributed data on different devices while protecting data privacy and saving significant network bandwidth [9]. However, models trained by the federated learning algorithm usually perform with lower accuracy than models trained by a standard centralized algorithm, especially when the training data are not independent and identically distributed (non-iid) on the edge devices. Zhu et al. provided a detailed analysis of the influence of non-iid data on learning model with FL algorithm [10].

FL consists of two steps, local training and global aggregation. In local training, the edge device downloads the model from the fog and computes an updated model using its local data. The fog server then collects these updated models mainly by averaging. FL can be used between fog and cloud also. In this case, the models’ parameters will typically be aggregated in the cloud by federating method and then distributed between the fog servers. The aggregation time in fog servers and cloud servers could be different, and this is an essential hyperparameter for deploying FL in edge, fog, and cloud computing [11,12].

The terms cross-silo and cross-device are two reflections of real-world usage and various Fl solutions for IoT applications that can be used in collaborative PM. Cross-silo can be related to FL between fog and cloud in PM applications, where the fog can be assumed as some companies that their PM data are geographically distributed, and the cloud can be assumed as a related data center considered by the specific field of work of these companies. Cross-device is the most commonly used FL setting in which clients are identified as resource-constrained IoT edge devices and can be used between edge and fog computation. This approach can facilitate remaining useful life prediction of an asset inside a manufacturing company by taking advantage of edge data analytics [13].

Models trained in federated learning usually perform worse than models trained in a centralized mode, especially when the training data are non-iid across local devices. This is very common in PM applications due to various anomalies at edge devices and human interaction in performing maintenance and reporting. Another issue in distributed statistical analysis, especially in PM, can be Simpson’s paradox. It occurs when data distribution causes some specific subgroups to have different trends of dependent and independent variables compared to the aggregated data, which is likely in PM. These issues need to be addressed in federated learning for collaborative PM, e.g., developing techniques for handling non-iid data distribution and proposing a kind of algorithms to detect the Simpson’s paradox at the edge and cloud levels.

### 1.1. Motivation

The cost of transferring all the PM raw data stored in the cloud and creating a centralized model is very high and violates privacy between organizations. The centralized models are more accurate than the distributed models. Still, the motivation of this paper is to find a distributed model that benefits from low data transfer to the cloud, reduces communication workloads, and has a comparable accuracy in predicting failure with centralized models.

**Figure 2:**
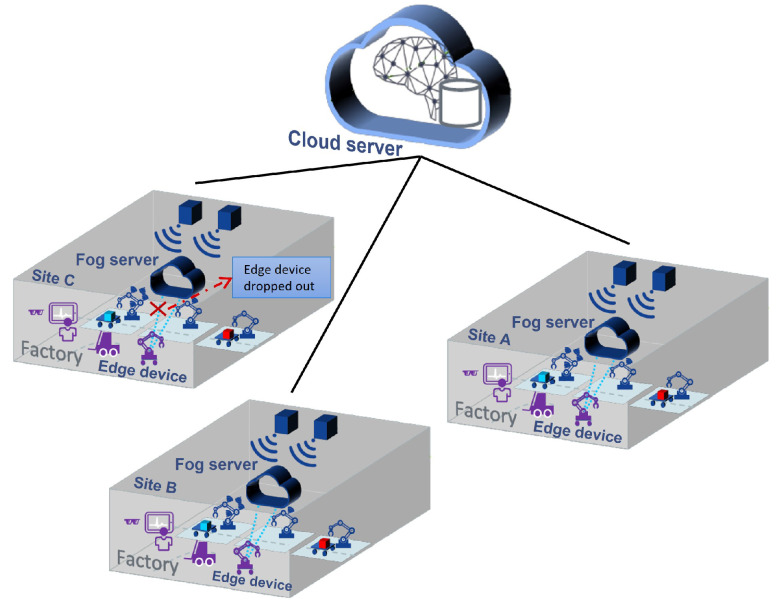
Proposed collaborative PM at the edge, fog, and cloud level.

In edge-level FL scenarios for PM, which involve heterogeneous devices and various failures, there may be clients who do not have sufficient computational resources to train the global model. Model aggregation and global model accuracy can be degraded due to these clients, which delays the training process. This situation may interrupt the collaborative training process, which is too costly in predictive maintenance scenarios. The proposed collaborative PM at the edge, fog, and cloud level is illustrated in Figure 2. Edge devices in each factory contribute to making a local model at the fog level, and then the global model is built from the model’s parameters in the fogs. The standard FL algorithm does not provide any predictions for limited communication resources. As a result, the release of a more evolved global model to clients may be delayed, and the system may not be able to predict failure before it occurs.

In order to use a simple and accurate ML model for collaborative PM scenarios, support vector machine (SVM) and long short term memory (LSTM) models are chosen to be treated in a federated manner. SVM is a non-probabilistic classifier that is suitable for classification and regression analysis. It separates data across a decision boundary such that the distance between the nearest sample and the decision boundary is maximal [14]. The effectiveness of SVM mainly depends on how the kernel and soft margin parameters are defined. The kernel function can determine whether the SVM is linear or non-linear. In this work, an SVM with a linear kernel is used in a federated manner to have the advantage of fast response in the communication rounds. LSTM is a recurrent neural network capable of learning long term dependencies between time steps of sequence data [15]. This capability helps process time series data flow in predictive maintenance applications. In this work, an LSTM with a random weighted connection of each cell is used in a federated manner, reducing the model’s dimensionality for the transition between different layers.

### 1.2. Contributions

1. This work proposes two federated models (FedSVM and FedLSTM) for collaborative PM, which provide a distributed model at FL edge devices. A communication graph describes the communication between the edge devices and the local server in each factory. This is achieved by activating these models in asynchronous mode. The server does not need to wait to collect parameters and can perform with comparable results to a centralized algorithm.
2. FedSVM utilizes a federated support vector machine (SVM) model in each edge device to classify PM strategies. It could predict labels that mentioned the need to do maintenance for one asset as an edge device at the cloud level without violating privacy. In preprocessing of the data before they are fed to the model, a moving average strategy has been implemented, which causes FedSVM to use a kind of memory inside its process. This federated method is reliable and fast enough for online applications such as PM.
3. FedLSTM utilizes a federated long short term memory (LSTM) model in each edge device to predict the absolute values of an asset’s RUL. This method is useful for learning from sequence data in each edge device. By applying the moving average strategy, the number of consecutive blocks in FedLSTM is reduced compared to without it, which significantly affects the training time of the model at the fog level.
4. These methods ensure that edge devices only exchange model parameters with the fog servers and the fog servers send them to the cloud server for aggregation. As well as they help speed up the local model convergence time because edge devices are sometimes unable to quickly and instantly access the cloud server, leading to delays in exchanging model updates.
5. FedSVM and FedLSTM are evaluated against a case study of RUL prediction for CMAPSS in a simulated collaborative PM. CMAPSS is a very well-known and benchmark dataset in RUL prediction. The results of these federal methods are compared with centralized approaches to model performance, communication resource utilization, and model convergence time.

The remaining of the paper is organized as follows. First, a brief review of the related research is presented in Section 2. Then, the federated learning system model and interaction between the different layers (edge, fog, and cloud) are discussed in Section 3. Section 4 is dedicated to explaining the details of FedSVM and FedLSTM architectures. Subsequently, Section 5 gives the structure of the performance evaluation of FedSVM and FedLSTM, and Section 6 analyzes the performance of the models and presents the experimental results. Finally, Section 7 concludes the paper and provides future research.

## 2. Related Work

Edge and fog computing are being increasingly used to deploy distributed ML algorithms, especially in resource-constrained environments [16,17,18]. Kay Bierzynski et al in [19] discuss four possible approaches for distributing the workload among Edge, Fog, and Cloud levels. Developments and challenges are also highlighted in this paper for implementation in hardware, machine learning, security, privacy, and communication. In general, this section is divided into two subsections. ML at the edge, fog, and cloud levels includes most related works on FL and optimization algorithms, distribution strategies, and hierarchical FL [9,10,11,12,20,21,22,23,24,25,26,27,28,29]. The second subsection, DML for collaborative PM, contains most related works that use FL in PM applications and distributed ML for collaborative PM scenarios [13,30,31,32,33,34,35].

### 2.1. Machine Learning at the Edge, Fog, and Cloud Levels

Centralized cloud computing is an ideal data integration solution to enable shared PM. However, due to industrial competition and data privacy, the manufacturing sector will not be satisfied with sharing its production data between companies. Due to legal restrictions, an organization may sometimes be reluctant to centralize its asset failure data collected from multiple production sites. Furthermore, the cost of centralizing all row data in a cloud is too high and not affordable. Thus, edge and fog computing offer a potential solution for the manufacturing industries to put isolated data islands together to teach better models while protecting their business intelligence.

Designing a system that enables the efficient distribution of machine learning is challenging because each algorithm has a distinct communication pattern. DML is a growing system with different solutions that differ in architecture, algorithm, efficiency, and performance [20].

Wang et al. have considered, in [21], the problem of learning model parameters from data distributed across multiple edge nodes without sending raw data to a centralized location such as fog or cloud. They introduced an algorithm based on distributed gradient descent to train the models, including SVM models, convolutional neural networks (CNNs), K-means, and linear regression. They analyzed the convergence rate of the proposed gradient-descent-based algorithm for distributed learning and showed that this convergence rate incorporates non-iid data.

Due to the rapid increase in FL research, several review papers have been published in this area. A comprehensive survey on FL and analysis it from five aspects: data partitioning, privacy mechanism, machine learning model, communication architecture, and systems heterogeneity are given in [22]. The key communication challenges of FL applications in IoT and edge devices are discussed in [23,24].

There are several algorithms for FL in distributed optimization. Most of these algorithms have been evaluated and compared in [25], including FedAvg, FedProx, CO-OP, and federated stochastic variance reduced gradient (FSVRG). The FedAvg algorithm works by running the training task on the edge devices, where they share an overall model with the central server that is an average of all the parameters.The FSVRG algorithm’s goal is to perform one full gradient computation centrally on fog or could, followed by many distributed stochastic updates on each edge device which is performed by iterating through a random permutation of the local data [26].

CO-OP proposes an asynchronous approach and merges any received edge model with the global model. Contrary to FedAvg, merging an edge and a global model is performed via a weighting scheme based on the models’ age difference. Another algorithm proposed for FL is called FedProx, which is similar to FedAvg. FedProx makes simple changes that allow for better performance and better heterogeneity. The reason behind this is that the different edge devices used for FL often have their own limitations, so it would not be ideal or realistic to expect all devices to do the same amount of work [27].

Different strategies for model aggregation and hierarchical FL are essential issues in a distributed system at the edge, fog, and cloud levels. Lumin Liu et al. proposed the hierarchical FL based on FedAvg for distributed systems [28]. They tried to formulate an optimization problem based on the number of aggregations at the fog and cloud levels compared to the number of iterations at the edge level. Based on their proposed architecture, this model can be trained faster and achieve better communication efficiency. Another study proposed a hierarchical FL to minimize training loss and latency by formulating an optimization problem of edge aggregation interval control and time allocation [29].

### 2.2. DML for Collaborative PM

Research shows that fog computing which offers more computation capability than edge devices can be exploited to implement ML algorithms for PM applications.

FL enables a collaborative model for PM at the edge level on cross-company data for different manufacturing sites or even distributed across multiple organizations. This method aims to exchange failure pattern data about one specific asset without sharing the raw data that can be considered as sensitive commercial data. The authors of ref. [30] proposed a new distributed PM algorithm based on FL and blockchain mechanisms.

One of the issues of using FL in the PM application is that some edge devices may not have sufficient computational resources to train the global model on time. These clients can cause delays in model aggregation and even disconnect during a training iteration and prevent other edge devices from efficient collaborative learning of the failure pattern from each other. To solve this issue, [13] proposed the Split Pred framework for collaborative PM, which provides a cross-device FL to implement reliable model training at edge devices.

A real-time fault detection system for edge computing was proposed in [31]. They used a two-layer architecture with a real-time fault detector on single-board computers, developed based on an LSTM recurrent neural network executing on the backend. Baotong Chen et al. proposed a system architecture for edge-based PM applications in IoT-based manufacturing. They showed that distributed learning in edge computing provides some advantages in terms of latency response for edge controlling and bandwidth optimization. A cooperation mechanism between edge, fog, and cloud computing is discussed to demonstrate the functionality of edge, fog, and cloud-based resources [32].

Ning Ge et al. in [33] have presented an empirical study on failure prediction in the production line based on FL. They have developed a federated SVM algorithm for the horizontal FL scenario and a federated random forest algorithm for the vertical FL scenario. They have also analyzed the effectiveness of FL in comparison with centralized learning. Their results reveal that the distributed FL algorithm can replace the centralized algorithm for failure and maintenance prediction.

One principle in predictive maintenance is anomaly detection, and some research has been done to deploy FL algorithms, mainly in this field. For instance, the authors in [34] introduced a novel FL algorithm for the LSTM framework and evaluated it on anomaly detection of sensors behavior in smart building applications. They proposed an FSLSTM network which consists of a local LSTM model that runs on sensor edge devices and a global model on fog that aggregates the weights, updates the parameters, and distributes them between the sensor edge devices. Their results show that this method converges twice as fast as the centralized LSTM model in the training phase. Similar work on anomaly detection is [35] in which authors proposed a novel communication efficient FL algorithm for sensing time-series data in distributed anomaly detection applications. They offered an attention mechanism-based convolutional neural network long short-term memory (AMCNN-LSTM) model to detect anomalies accurately. This model captures the most important features with the use of a CNN and then passes data to an LSTM, which predicts the future time-series data.

For ease of resource following, a concise summary of the related research is provided in Table 1.

## 3. System Model

In this section, we describe the framework of FL over wireless communication in different levels of edge, fog, and cloud layers. We will discuss the network model, federated learning process, learning model, and how to fit the idea of FL on SVM and LSTM models.

### 3.1. Network Model

As depicted in Figure 2, we considered a general FL-supported wireless multi-agent network between fog server and *N* distributed edge devices, denoted as the set N={1,…,N}. The fog server is directly connected to the cloud server through a wireless link with the nearest base station. The fog server in each factory is also equipped with computational resources to provide communication and computation services to the edge devices. The edge devices could be some similar assets in a factory site, and communicate with the fog server for an FL task via a wireless link. We assume that each *i* asset on a factory site collects measurement data and has information about labeled training samples, such as RUL information of one asset with its sensors measurement. This dataset is denoted as $D_{i}={\left\{\xi_{i,l}\right\}}_{l=1}^{D_{i}}$, which ξ represents the lth training sample at edge device i,∀i∈N. The whole dataset in each factory is the union of its edge devices datasets $D={⋃}_{i\in N}D_{i}$. We consider two machine learning models (SVM and LSTM) over this wireless network between fog server and edge devices in different factory sites. The fog server and edge devices collaboratively build shared SVM and LSTM models for predicting the labels. These shared models are trained by exchanging model parameter information while keeping all the data locally at the edge devices. The global shared model will be made by aggregating all the model parameters from fog servers.

### 3.2. Federated Learning Process

In an FL algorithm, a specific ML model is trained in a distributed manner among some edge devices and then aggregated in a server (i.e., fog server). The goal of this kind of process is to find a fog level model parameter $w\in R^{d}$ in each factory with the objective of minimizing a loss function f(w) on the whole dataset of that factory site. *d* is the dimension of the parameters and should be the same for all factories. The fog and edge level learning objective of the network can be expressed as follows, which is considered only for one factory site.

(1) $$\begin{array}{cc} \; & \min_{w}\left\{f\left(w\right)\right\}\\ f(w) & =\sum_{i=1}^{N}\frac{D_{i}}{D}f_{i}\left(w\right)\\ f_{i}\left(w\right) & =\frac{1}{D_{i}}\sum_{l=1}^{D_{i}}F_{i}\left(w,\xi_{i,l}\right), \end{array}$$

where $D_{i}=\left|D_{i}\right|$ is one of the data samples, $f_{i}\left(w\right)$ is the local loss function of edge device *i*, and $F_{i}\left(w,\xi_{i,l}\right)$ characterizes the loss of model parameter *w* on the training sample $\xi_{i,l}$. With these definitions, the main objective in the cloud is to minimize the global loss function $F_{G}\left(w\right)$ which is defined as the summation of all loss functions $f^{j}\left(w\right)$ in different factories, j,∀j∈M is the index number of a factory site.

(2) $$\begin{array}{c} F_{G}\left(w\right)=\sum_{j=1}^{M}f^{j}\left(w\right). \end{array}$$

The principal analysis in the cloud and fog layer is based on the federated averaging (FedAvg) Algorithm. The whole training process is periodical with an arbitrary number of communication rounds ($T_{G}$ is the number of global communication rounds between cloud and fog servers, and $T_{l}^{j}$ is the number of local communication rounds between edge devices and fog server in different factories). Each local communication round has a different number of iterations on edge devices *E*, defined as local epochs. The training process at the tth communication round is described as follows:

1. Broadcasting from cloud server: Cloud server broadcasts the global model parameter $w_{cloud}^{t}$ to all fog servers through a wireless link in the tth round.
2. Fog updating phase: All fog servers update their model parameters with the received parameters from the cloud.
3. Broadcasting from local fog server: Fog servers broadcast the updated model parameters $w_{fog,j}^{t}$ to all edge devices located at factory site number *j* through a wireless link.
4. Edge device updating phase: After receiving the fog level model parameter, each edge device i∈N in factory j∈M trains its local model by applying *E* epochs of a kind of optimization algorithms such as SGD and Adam. The iteration for SGD becomes
  (3) $$\begin{array}{c} w_{i,j}^{t+1}=w_{i,j}^{t}-\eta \nabla f_{i}^{j}\left(w_{i,j}^{t}\right), \end{array}$$
  where η is the learning rate and $\nabla f_{i}^{j}\left(w_{i,j}^{t}\right)$ is the stochastic gradient of local loss function in edge device *i* in factory *j*. After *E* epochs, edge device *i* uploads its update model parameter $w_{i,j}^{t+1}$ to the connected fog server *j*.
5. Aggregating on fog: After *E* iterations on the edge devices in each factory, once receiving all the local model parameters, the fog server aggregates them and obtains an updated model, which is known as a synchronous method.
  (4) $$\begin{array}{c} w_{fog,j}^{t+1}=\sum_{i=1}^{N_{j}}\frac{D_{i}^{j}}{D^{j}}w_{i,j}^{t+1}, \end{array}$$
  where $D^{j}$ is the whole data sample at factory site *j*. Another aggregation method is that when an edge device updates its model parameter in fog, the server in fog immediately creates an intermediate form of that agent’s parameters with its parameters and returns it to the agent for the next iteration. In this method, defined as asynchronous aggregation, the servers in the fog do not need to wait until they receive all the agents’ parameters.
6. Aggregating on cloud: The model parameter aggregation on the cloud happens once in a while. The number of communication rounds between cloud and fogs is much lower than the number of communication rounds between edge devices and fogs ($T_{G}<<T_{l}^{j}$). Therefore, when the cloud requests an update, a simple averaging with different weights ($A_{j}$) depending on the size of the factory is performed on all fog parameters
  (5) $$\begin{array}{c} w_{cloud}^{t+1}=\sum_{j=1}^{M}A_{j}w_{fog,j}^{t+1}, \end{array}$$
  where $\sum_{j=1}^{M}A_{j}=1$.

The complete description of edge, fog, annd cloud FL algorithm is shown in Algorithm 1.
**Algorithm** **1:** Federation on the edge, fog, and coud.
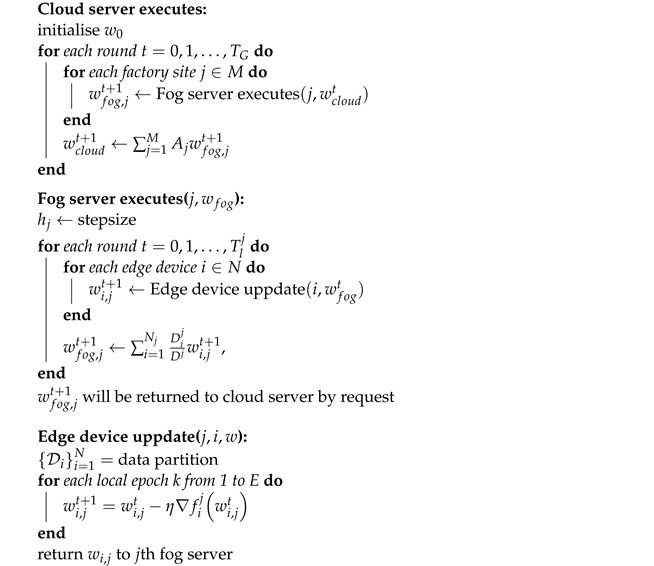


## 4. FedSVM and FedLSTM: Proposed Architecture

### 4.1. FedSVM

SVM is a supervised ML algorithm used to analyze data for classification. On PM applications, it could be used as an alarm notification to inform factory management about performing maintenance on one asset that is close to failure. The idea of FedSVM is first to find one or several hyperplanes at the fog level that separate and classify failure data from healthy data of all assets in a factory site and aggregate these hyperplanes at the cloud by using federated averaging. The SVM model in each asset could have the largest margin on a hyperplane separating the two groups. Still, the FedSVM wants to find the largest margin on a federal hyperplane on the cloud without sharing the local data from edge devices and, subsequently, fog servers.

**Figure 3:**
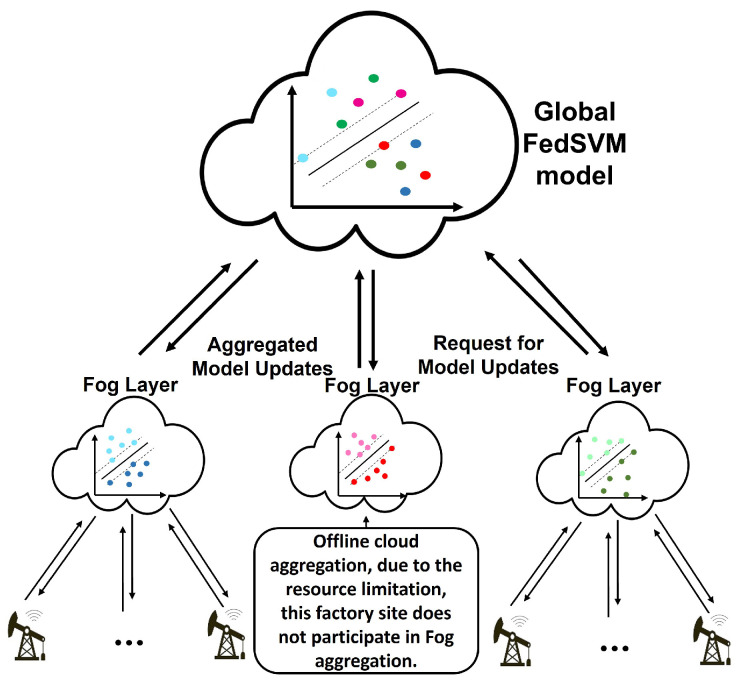
FedSVM architecture.

The FedSVM architecture is illustrated in Figure 3. In order to aggregate the FedSVM model at the fog and cloud level, we need to access the different edge devices’ support vectors and their intercepts. After pre-processing the data set from each device at each factory site, the faulty and healthy parts of the data can be separated from each other by setting a threshold, i.e., measurement data from a device as long as its RUL is greater than the threshold is assigned to +1, and the other part is assigned to −1. This enables us to define the same objective function at the fog levels, as shown in Equation (6).

(6) $$\begin{array}{c} f\left(w\right)=\frac{1}{D}\sum_{i=1}^{N}f_{i}\left(w\right)+{\lambda ||w||}_{2}^{2}\\ f_{i}\left(w\right)=\max\left(0,1-y_{i}w^{T}x_{i}\right), \end{array}$$

where $x_{i}$ is the features extracted from the dataset, and $y_{i}$ is the label related to the features for one edge device at one factory site. Due to the imbalance and non-iid data distributed on edge devices in PM applications, λ as a regularizer has been added to the local loss function. For updating the parameters at the fog levels, tho following gradient descent has been used:

(7) $$\partial f_{i}\left(w\right)=\left\{\begin{array}{cc} -y_{i}x_{i}\;\; & 1-y_{i}w^{T}x_{i}>0\\ 0\;\; & \mathrm{Otherwise} \end{array}\right..$$

By these definitions, the subgradient for f(w) at the fog levels becomes

(8) $$\begin{array}{c} \partial f\left(w\right)=\frac{1}{D}\sum_{i=1}^{N}\partial f_{i}\left(w\right)+2\lambda w. \end{array}$$

One of the advantages of using FedSVM is that it is very fast and has a low convergence time. Therefore it is very useful for online applications. The results of this method will be presented in Section 6.

### 4.2. FedLSTM

The LSTM is a kind of recurrent neural network (RNN) that has the ability to take feedback from multiple hidden layers in a specific way instead of one hidden layer like an RNN. The LSTM can manage some memory blocks to remember the input pattern at the beginning of the sequence [15], and it is beneficial for the prediction of temporal data such as PM applications.

**Figure 4:**
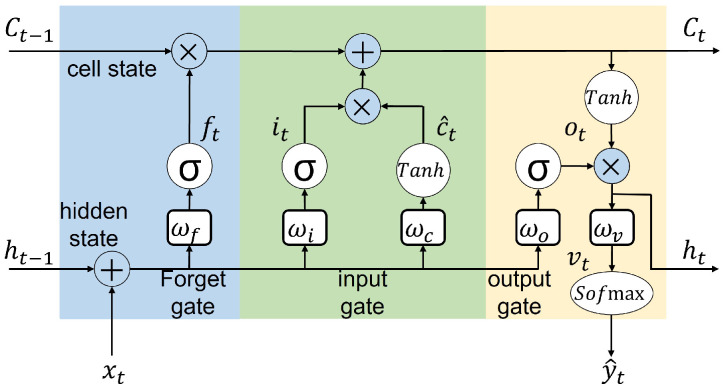
LSTM architecture and memory blocks.

The LSTM neural network consists of several memory blocks, each consisting of a memory cell and three types of gateways, as shown in Figure 4. Memory blocks are the main infrastructure of an LSTM, enabling the LSTM to learn how long it has to remember old time information when to forget, when to use new data, and how to generate output by Combining the old memory with new input.

The equations of the LSTM scheme are given as follows.

(9) $$\begin{array}{cc} \; & f_{t}=\sigma \left(w_{f}\left[h_{t-1,x_{t}}\right]+b_{f}\right)\\ \; & i_{t}=\sigma \left(w_{i}\left[h_{t-1,x_{t}}\right]+b_{i}\right)\\ \; & {\hat{c}}_{t}=\tanh\left(w_{c}\left[h_{t-1,x_{t}}\right]+b_{c}\right)\\ \; & c_{t}=f_{t}⊗c_{t-1}+i_{t}⊗{\hat{c}}_{t}\\ \; & o_{t}=\sigma \left(w_{o}\left[h_{t-1,x_{t}}\right]+b_{o}\right)\\ \; & h_{t}=o_{t}⊗\tanh\left(c_{t}\right), \end{array}$$

where $f_{t}$, $i_{t}$, and $o_{t}$ present the forget, input, and output gates, respectively, *w* and *b* are the corresponding weight and bias parameters for these gates, *c* is used for cell state, and *h* is the hidden state. Moreover, σ represents the sigmoid activation function, and ⊗ indicates the Hadamard product.

The standard LSTM is made by the number of sequential blocks. Each of them is the same as Figure 4 and sequentially connected. At the final block, a softmax function is used as a final activation function, given as follows for the prediction.

(10) $$\begin{array}{cc} \; & v_{t}=w_{v}h_{t}+b_{v}\\ \; & {\hat{y}}_{t}=\mathrm{Softmax}\left(v_{t}\right), \end{array}$$

By defining the cross-entropy loss function from the output of the LSTM block and computing the partial deviation of this loss function with respect to the weights and bias, the parameters of the model will be updated in a backpropagation process. after some iterations (epochs) on this backpropagation process, the parameters should be sent to the specific fog server for the fog aggregation. i.e., the gradient descent update rules for the forget gate are as follows.

(11) $$\begin{array}{cc} \; & w_{f}^{t+1}=w_{f}^{t}-\eta \frac{\partial J}{\partial w_{f}}\\ \; & b_{f}^{t+1}=b_{f}^{t}-\eta \frac{\partial J}{\partial b_{f}}, \end{array}$$

where ∂J is the derivation of the cross-entropy loss.

**Figure 5:**
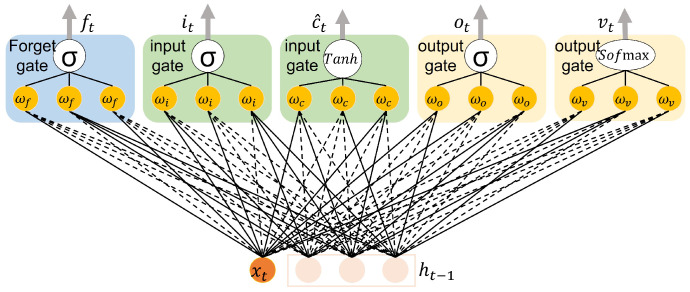
Random topology formation of FedLSTM.

Compared to the SVM model, the LSTM has a massive number of weight and bias parameters that force it to use higher bandwidth in the model distribution between edge, fog, and cloud levels and adds a computational delay to the system. Therefore, the conventional LSTM model is not proper for distributed learning in which the neurons in each memory block are fully connected. FedLSTM proposed a random topology formation of synapses used at each edge, fog, and cloud levels. This desired topology must first be designed in the cloud and the model configuration applied to all fog servers and edge devices. The random FedLSTM topology is shown in Figure 5, the dashed lines show the synapses removed from the model, and the solid lines represent the remaining synapses. This model will be distributed at the different edge, fog, and cloud levels, such as FedSVM in Figure 3.

## 5. Structure of Performance Evaluation

This and the following section investigate the efficiency of FedSVM and FedLSTM based on the model accuracy, convergence time, and communication resource usage in a collaborative PM scenario. For this, we need a dataset to analyze the efficiency of the model. Particularly in RUL prediction, CMAPSS [36] is a well-known and benchmarked dataset. Many types of research have been done on this dataset, which is helpful for the result comparisons of FedSVM and FedLSTM with centralized solutions.

### 5.1. Distributing CMAPSS for a Collaborative PM

C-MAPSS is a nonlinear dynamic model of a commercial turbofan engine implemented in the MATLAB/Simulink by NASA. Changing the input parameters in this simulation model makes it possible to simulate various degradation profiles in different engine conditions. 4 time series (FD001, FD002, FD003, FD004) with different fault modes were generated using these simulation tools. These dataset consists of multivariate time series and each of them is divided into training and testing subset.

FD001 has 100 test trajectories and 100 train trajectories, including one fault mode and one degradation (high-pressure compressor (HPC) degradation). FD002 has 260 test trajectories and 259 train trajectories, including six fault modes and one degradation (HPC degradation). FD003 has 100 test trajectories and 100 train trajectories, including one fault mode and two degradations (HPC degradation, Fan degradation). FD004 has 248 test trajectories and 249 train trajectories, including six fault modes and two degradations (HPC degradation, Fan degradation).

**Figure 6:**
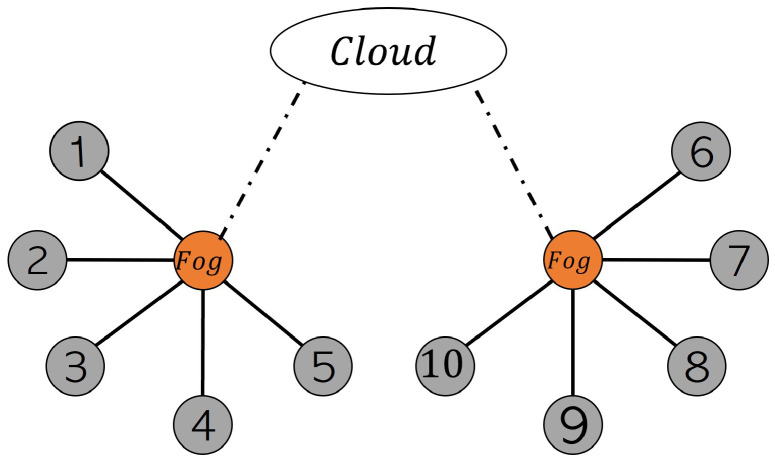
Undirected graph of communication between edge devices and fog servers for synchronous FL.

Each time series consists of 21 sensor observations, three operating settings, a trajectory-id, and a cycle count, which the RUL of an engine is estimated from it in terms of the number of operation cycles before the engine runs to failure. The goal is to predict the RUL based on the time series data by the model at the cloud level, which was trained by the federated aggregation from the fog servers and, subsequently, edge devices. The whole CMAPSS is used throughout the experiments. i.e., time series FD001 is distributed equally among ten edge devices located in two different factory sites for participating in a collaborative PM scenario. Each edge device can train the global model (FedSVM or FedLSTM) by using its local time-series data. The graph connectivity of the edge devices in two factory sites is shown in Figure 6. FedSVM and FedLSTM based on Algorithm 1 is performed in different edge, fogs, and cloud layers of this undirected graph. This is a synchronous federated learning method in which fog and cloud have to wait to collect all the parameters from the edge devices.

**Figure 7:**
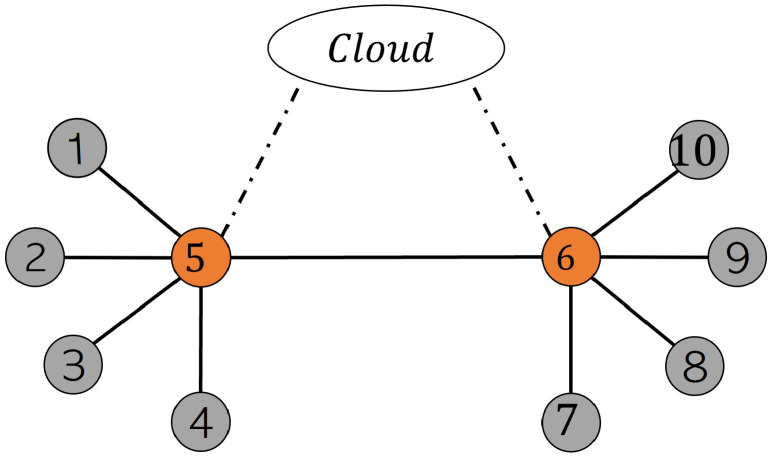
Undirected graph of communication between edge devices and fog servers for asynchronous FL, edge devices 5 and 6 play the fog roll.

Another type of graph connectivity is shown in Figure 7. Edge devices 5 and 6 can play a role similar to fog servers in this configuration. This configuration is known as asynchronous federated learning, in which each edge device can share its parameter with its neighbors, which act as fog here. The difference between the asynchronous algorithm and algorithm 2 is that the fog agents perform a simple federal averaging when they receive a new parameter from one edge device and immediately return the recent update to the specific edge from which it received the last parameter. It is assumed that fog servers in different factories can communicate with each other. With this method, if some edge devices cannot participate in fog aggregation due to resource constraints, the fog model can continue to operate appropriately. The parameter sends from fog servers to the cloud by request from the cloud.

By defining a doubly stochastic matrix as an undirected graph $G_{A}\left(E,V\right)$ with vertex set V and edge set E, it could be possible to manage the gradient descent over a massive network connection. The doubly stochastic matrix A is defined as follows.

(12) $$\begin{array}{cc} \; & A\in R^{d\times d}:a_{ij}\geq 0,\forall i,j,\;A1=1\;\mathrm{and}\;A^{T}1=1, \end{array}$$

where (i,j)∈E iff (j,i)∈E and $a_{ij}\geq \eta$ for some small positive η and set of neighbors of vertex *i* is $N_{i}=\left\{j\in V|\left(i,j\right)\in E\right\}\cup \left\{i\right\}$. For the undirected graph shown in Figure 7, which has been used for the collaborative PM scenario, the doubly stochastic matrix is defined as follows to see which nodes are directly connected.

$$A=\left[\begin{array}{cccccccccc} 1 & 0 & 0 & 0 & 1 & 0 & 0 & 0 & 0 & 0\\ 0 & 1 & 0 & 0 & 1 & 0 & 0 & 0 & 0 & 0\\ 0 & 0 & 1 & 0 & 1 & 0 & 0 & 0 & 0 & 0\\ 0 & 0 & 0 & 1 & 1 & 0 & 0 & 0 & 0 & 0\\ 1 & 1 & 1 & 1 & 1 & 1 & 0 & 0 & 0 & 0\\ 0 & 0 & 0 & 0 & 1 & 1 & 1 & 1 & 1 & 1\\ 0 & 0 & 0 & 0 & 0 & 1 & 1 & 0 & 0 & 0\\ 0 & 0 & 0 & 0 & 0 & 1 & 0 & 1 & 0 & 0\\ 0 & 0 & 0 & 0 & 0 & 1 & 0 & 0 & 1 & 0\\ 0 & 0 & 0 & 0 & 0 & 1 & 0 & 0 & 0 & 1 \end{array}\right]$$

### 5.2. Data Preprocessing

#### 5.2.1. Feature Selection

The time series raw in the CMAPSS dataset should be analyzed and arranged in the same size and model for distribution between the edge devices. Features that remain constant at all stages of time can have a negative impact on model training. The variability of features of the dataset during failure was measured by the processability function and has removed some sensor data which have zero profitability. Therefore, 14 sensor measurements out of 21 sensors and two operating settings out of a total of three are used as the raw input features. Then the training predictor was normalized to have a zero mean and unit variance. In order to focus the FedLSTM model more on the part of the data that the engines are most likely to fail, a clip of the responses has been made at the threshold of 100-cycle, and it is performed over all edge devices.

#### 5.2.2. Moving Window Strategy

The input data to both models are in a 2D format like an image where one dimension represents the sequence length, and the other represents the number of features. However, the length of the time series data varies for each engine. Therefore, a fixed length of the data sequence was extracted to make it compatible with the FedSVM and FedLSTM training models.

The FedSVM is very fast and takes very little time to train, but it has no capabilities to learn the temporal dependencies. Therefore, we added a kind of memory to the SVM model by using the moving window strategy that creates multiple input and output mapping while preserving the stochastic dependencies between the predictions. Moreover, the moving window strategy can behave very well on the LSTM model for complex dependencies since this method makes it possible to decrease the LSTM sequential blocks and subsequently FedLSTM computational time.

**Figure 8:**
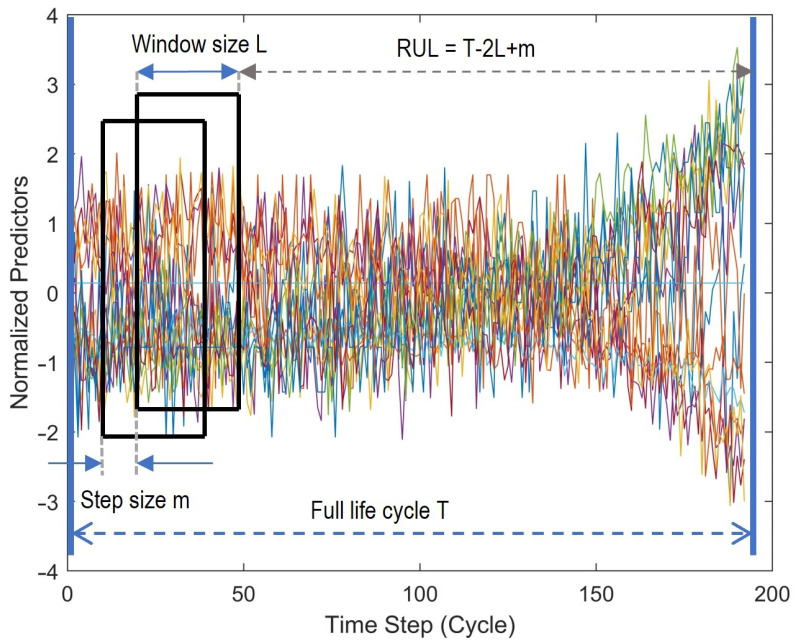
Moving window strategy over sensor measurements of an engine.

The idea of the moving window is simple, a window with fixed values of window length and stride, sliding over all the features data. Window length describes the length of the time series data as a short memory used for RUL prediction at the end of the time stamp, and stride is the value of shift between two consecutive windows. The corresponding output for each sequence is the RUL at the last time-stamp of that sequence. The sensor measurements of one engine and the moving window strategy are illustrated in Figure 8.

### 5.3. Evaluating Metrics

Two popular metrics for evaluating the RUL prediction results based on FedLSTM have been used in this study. Root mean square error (RMSE) and scoring Factor (SF). RMSE is used to measure the deviation between the observation and actual values. This is a standard evaluation indicator for error prediction.

(13) $$\begin{array}{c} \mathrm{RMSE}=\sqrt{\frac{1}{\kappa }\sum_{i=1}^{\kappa }{\left({\hat{Y}}_{i}-Y_{i}\right)}^{2}}, \end{array}$$

where κ is the total number of test samples, which is defined based on the number of input matrices coming from the moving window, ${\hat{Y}}_{i}$ is the prediction value of RUL of the ith turbofan engine, and $Y_{i}$ is the actual value of the ith turbofan engine.

Another evaluating metric is SF, which is an asymmetric function. It is used to evaluate the prediction model based on early predictions and late predictions. The function achieves a higher score when the RUL prediction value is less than the actual value. To avoid serious consequences due to delayed prediction in PM application, it tends to predict early rather than late.

(14) $$\begin{array}{c} \;\mathrm{SF}\;=\left\{\begin{array}{c} \sum_{i=1}^{x_{n}}e^{\left(\frac{{\hat{Y}}_{i}-Y_{i}}{13}\right)}-1,\;\;\;\;\;\;{\hat{Y}}_{i}-Y_{i}<0\\ \sum_{i=1}^{x_{n}}e^{\left(\frac{{\hat{Y}}_{i}-Y_{i}}{10}\right)}-1,\;\;\;\;\;\;{\hat{Y}}_{i}-Y_{i}\geq 0 \end{array}\right.. \end{array}$$

The model with lower RMSE and SF values has higher efficiency and effectiveness. For evaluating FedSVM, the following accuracy formula has been used

(15) $$\begin{array}{c} \mathrm{accuracy}=\frac{\mu_{P}+\mu_{N}}{\mu_{P}+\mu_{N}+\Gamma_{P}+\Gamma_{N}}, \end{array}$$

where $\mu_{P}$, $\Gamma_{P}$, $\mu_{N}$, $\Gamma_{N}$ are the number of true positives, false positives, true negatives, and false negatives, respectively.

## 6. Experimental Results and Discussion

The reported experimental results on FedSVM and FedLSTM are averaged by ten trials to reduce the effect of randomness. All the experiments are performed in python on a Laptop with Intel Core i7–2.3 GHz CPU, 32-GB RAM, and NVIDIA RTX A3000 GPU. All the edge devices, fog servers, and cloud servers work as virtual workers inside the python script and collaborate based on the proposed algorithms and two communication topologies. The only library used is Numpy to manage the database and scientific computing. All the gradient descent methods, algorithms, and configuration has been implemented from scratch. FedSVM and FedLSTM algorithms based on python are available at Github [37].

The fedSVM and FedLSTM models are analyzed based on the proposed communication topologies shown in Figure 6 and Figure 7, and subsequently on synchronous and asynchronous federated algorithms.

### 6.1. FedSVM results

**Figure 9:**
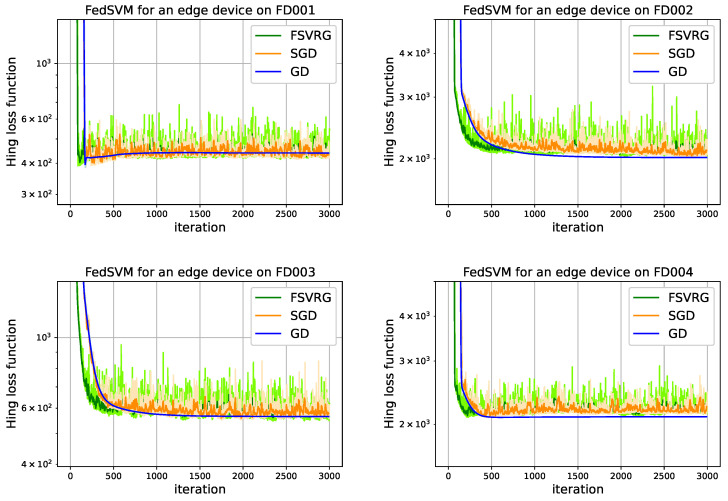
Convergence time of syncronous FedSVM based on the communication of Figure 6 with different optimizer.

Figure 9 depicts the descending hinge loss function and convergence time of one edge device on topology Figure 6 with the model optimizer (*GD*, *SGD*, and *FSVRG*). The convergence time of a predictive model represents the number of iterations required at edge devices to converge to an optimal loss function value. In FedSVM, each iteration executes once time at edge level, and then the parameters are updated and sent back to the fog servers. The number of iterations of a model optimizer at the edge level is called epoch. When epoch is equal to one, it means that the number of iterations is equal to the number of communication between the edge devices and the fog servers. Learning rate has a high impact on convergence time. Therefore, this parameter plays a crucial role in distributed learning. Because it not only consumes the computing resources of edge devices but also involves several message exchanges between them. The results in Figure 9 show the fastest convergence on synchronizing FedSVM with a learning rate equal to 0.01, as can be seen, synchronous FedSVM can converge on different time-series of CMAPSS dataset only with 500 iterations. These features make FedSVM appropriate for distributed learning.

The cloud aggregation executes three times during the training process. Cloud aggregation can be performed randomly when the channel load is low. Then the final model in the cloud is used to predict the labeled RUL on the testing subset related to the different time-series data. In FedSVM, the RUL prediction is restricted to a new label in which less than TH as a threshold is “1”, and more than TH is “−1”. In this way, two classes are generated, and the FedSVM classifier can be executed at the edge devices. This threshold was set at 50, which means that the edge machine needs maintenance when its RUL is less than 50 cycles. Based on the moving window strategy, 17 arrays of time-series data have been selected as features. If the length of the window is 35, then a 35 by 17 array of measured signals with normalized values from 0 to 1 is generated as an input to the model. Each of these arrays is 35×17=595, and a new entry into each vector of value “1” is also appended to them, which enables the transformation $w^{T}x_{i}$ to be affine rather than strictly linear.

**Figure 10:**
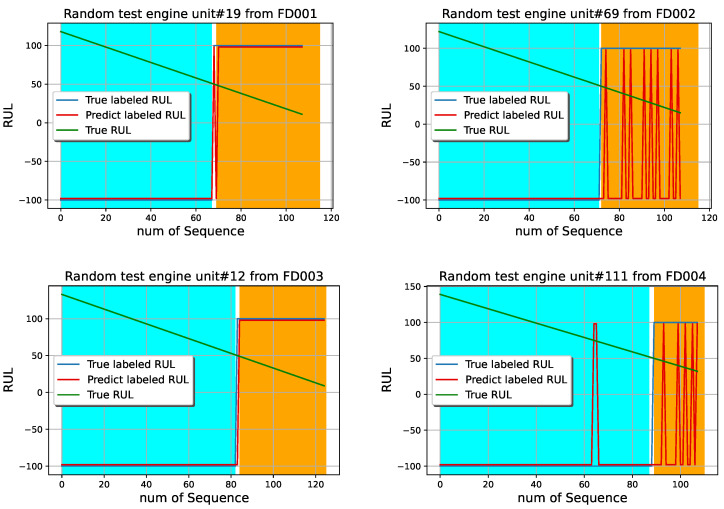
Four example of labeled RUL predictions for the testing engine based on the synchronous FedSVM model.

Furthermore, the labeled RUL prediction based on the synchronous FedSVM of the testing engine units in different time series of CMAPSS are shown in Figure 10. One example randomly selected out of whole testing engine units, whose unit numbers are 19, 69, 12, and 111 respectively from FD001, FD002, FD003, and FD004, are presented for demonstration. The cyan band shows the safe operation of the engine, and the chrome yellow band indicates that the engine needs to do maintenance. The green line is the true RUL, the blue line is the labeled RUL, and the red line is the prediction of the labeled RUL. Therefore blue and red lines have only two values, “−100” demonstrates that the engine is working on the safe operation side, and “100” notes that the engine needs to do maintenance. It can be observed that the synchronous Fedsvm can detect the need to do maintenance very well, even on the FD002 and FD004, if the machine will be stopped after the first indication of the red line for doing maintenance. FD002 and FD004 time series prediction is more complicated than FD001 and FD003.

Table 2 summarizes the performance of synchronous FedSVM, the duration of the training process, which shows how much this algorithm is fast, is notified by the runtime, and the final accuracy which has been calculated based on the (15), is illustrated in this table. It can be observed that with the proposed synchronous FedSVM, even though the data are distributed at the edge devices and the model is only shared between the edges, the average accuracy of the algorithm is more than 85%.

The performance of asynchronous FedSVM based on the undirected graph communication in Figure 7 is summarized in Table 3. It can be observed that even though, with this algorithm, none of the fog servers need to wait to receive all the update parameters from all edge devices, the average accuracy is acceptable, and it is close to the accuracy of synchronous FedSVM. The asynchronous FedSVM is implemented with GD and SGD optimizer, and due to the limitation of asynchronously updating the parameters from the edge device, the FSVRG optimizer is not implemented.

### 6.2. FedLSTM Results

**Figure 11:**
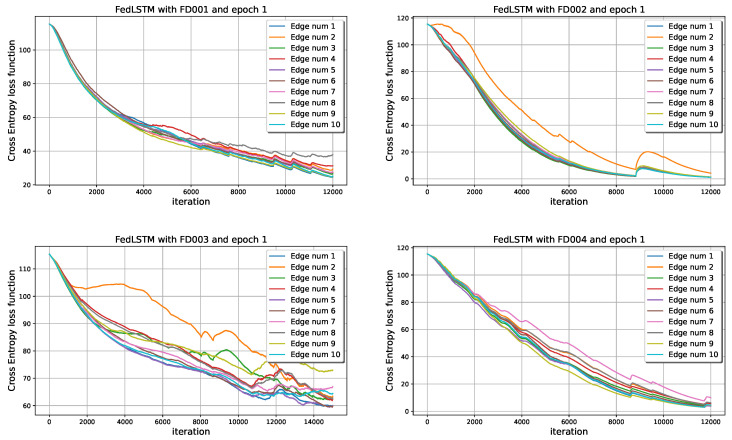
Convergence time of syncronous FedLSTM based on the communication of Figure 6.

Each edge device’s 2D format input data from the moving window strategy is fed to the LSTM block, as shown in Figure 4. The process is that 25 of these blocks are sequentially arranged, and the whole 2D input data at each time is equally divided between these blocks. The final block in this sequence led to a softmax activation function to predict the high probability of RUL value related to the whole input 2D data. The results in Figure 11 show the convergence of synchronous FedLSTM with only one iteration at the edge level and 100% connection of neurons. Non-iid data have a significant impact on the convergence of distributed learning. As it turns out, edge number 2 in FD002 and FD003 has a different anomaly that does not let it decrease the cross-entropy function at the same rate as other agents. One of the solutions for this problem is to increase the number of iterations at the edge level (epoch), which causes all cross-entropy functions to have the same convergence rate. The effect of increasing the epoch number on the model’s accuracy will be shown later.

**Figure 12:**
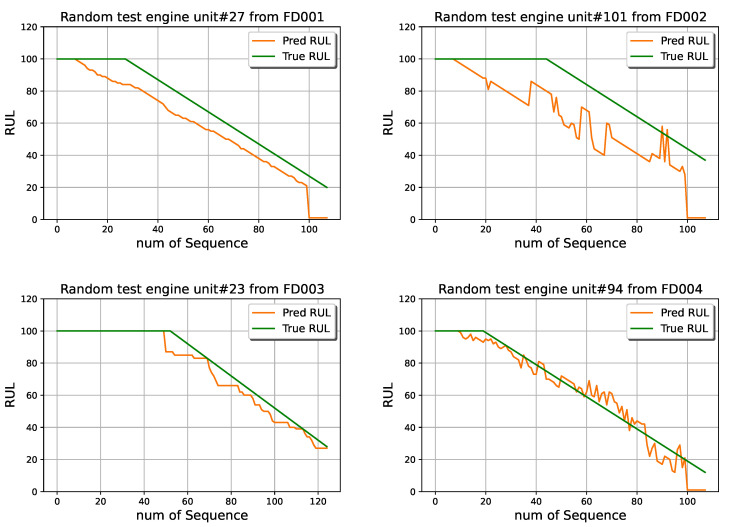
Four example of RUL predictions for the testing engine based on the synchronous FedLSTM model.

The RUL prediction results based on the synchronous FedLSTM of the testing engine units in different time-series of CMAPSS are shown in Figure 12. One example randomly selected out of whole testing engine units, whose unit numbers are 27, 101, 6, and 94 respectively from FD001, FD002, FD003, and FD004, are presented for demonstration. In predictive maintenance applications, factory owners desire higher model prediction accuracy in regions where the RUL value is small. As a result, RUL values above 100 are clipped to 100 for all testing and training parts in the FedLSTM model. It can be observed that with the FedLSTM model, even without transferring a massive amount of measured data between edge, fog, and cloud levels and violating privacy, predictive accuracy is acceptable on all time-series datasets, especially when edge devices are close to failure.

The two evaluation metrics used chiefly for analyzing the results are RMSE and SF, explained in Section 5.3. The whole training process for synchronous FedLSTM has been done with epoch numbers from 1 to 4, and the evaluating metrics have been calculated. These results are summarized in Table 4. furthermore, the performance of asynchronous FedLSTM based on the undirected graph communication Figure 7 is summarized in Table 5. The number of communication cycles between edge, fog, and cloud is the same in all experiments, and only the number of iterations in each agent has been changed.

The deep learning algorithm obtains good results in centralized RUL prediction in the CMAPSS dataset. To compare the proposed FedSVM and FedLASTM model with centralized prediction, some best predictions based on deep learning are shown in Table 6. By comparison of the FedLSTM results with other prediction methods in Table 5, it can be confirmed that the performance of the proposed FedLSTM in PM applications has comparable efficiency to the conventional centralized approaches in terms of prediction accuracy.

**Figure 13:**
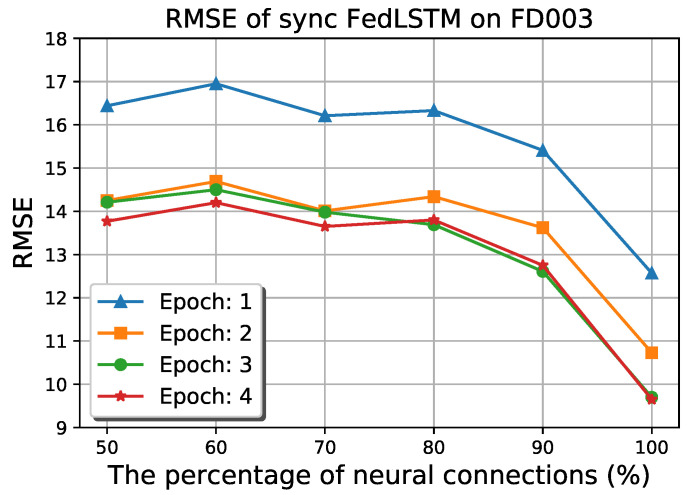
Results of random neural connection on synchronous FedLSTM.

Conventional LSTM models in a distributed system suffer from a large number of model parameters that must be passed between edge, fog, and cloud levels. Therefore, FedLSTM proposed the random topology formulation of neural connections on each gate. Figure 13 depicts the RMSE of synchronous FedLSTM on FD003 under the different percentages of neural connectivity. It can be observed that only 30% of the model accuracy has been lost by reducing the number of synapse connections to half, and on the other hand, the bandwidth usage and the training time are also decreased.

### 6.3. Model Aggregation Analysis

Image classification tasks are considered for model aggregation analysis with FedSVM, and the Modified National Institute of Standards and Technology dataset (MNIST dataset) is used. Due to the binary classification of FedSVM, Two highly similar labels are selected from the 10 hand-written classes, labels 1 and 7. A hierarchical FL with ten edge devices, two fog servers, and a cloud server has been considered, similar to two topologies of undirected communication graphs demonstrated in Figure 6 and Figure 7. The distribution of training data is the main issue in the FL algorithm. Therefore we have considered the following two distribution cases and distributed the training data regarding labels 1 and 7 between the edge devices.

1. Edge-iid: The training data from labels 1 and 7 are identically distributed between the ten edge devices.
2. Edge-non-iid: The training data of label 1 are distributed among edge numbers 1 to 5 under one fog server, and training data of label 7 are distributed among edge numbers 6 to 10 under another fog server.

The experiments of FedSVM for the proposed aggregation strategy are done with the MNIST dataset under two distribution cases. Table 7 summarizes the performance of these experiments, including the test accuracy and run-time of calculation. It can be observed that the test accuracy of label prediction at the cloud with any optimization methods and different conditions of iid, non-iid, synchronous, and asynchronous is around 97%. This accuracy, compared with other similar proposals’ accuracy [28,29], shows this proposed architecture’s advantage. The test accuracy of MNIST prediction in [28] is around 95%, and in [29] is about 93%.

## 7. Conclusions

Distributed ML algorithm over edge devices and their cooperation with fog and cloud is a fast-growing research area with many challenges and opportunities, especially in PM applications. Using federated ML algorithms has been shown to improve not only the privacy and security of edge devices’ data but also communication efficiency and system response time for real-time applications. In this article, two distributed models known as FedSVM and FedLSTM were proposed to enable local edge devices within an FL algorithm, to collaboratively train a global model at the cloud level in the context of collaborative PM application. FedSVM model was analyzed based on two different communication topologies and tested for convergence time and accuracy. FedSVM was found to be very fast in training and suitable for distributed online applications in predicting the time to do maintenance. On exact RUL prediction on distributed systems, FedLSTM with the random connection between the neurons has been proposed and analyzed based on two different communication topologies with synchronous and asynchronous algorithms and tested on the CMAPSS dataset for convergence time and model accuracy. Comparison with state-of-the-art research on centralized RUL prediction with CMAPSS revealed that the utilization of FedSVM and FedLSTM results are comparable with centralized algorithms and furthermore improving not only the privacy and security of edge devices but also communication efficiency and system response time for real-time applications. The FedSVM model has also been used for digits classification of the MNIST dataset and shows that the aggregation strategy is general and can be used with other learning algorithms.

Improving the model aggregation algorithm to deal with heterogeneous hardware at the edge level, non-iid data and Simpson’s paradox, which are popular in PM applications due to the different anomaly occurs at edge devices are other research topics for future.

## Figures and Tables

**Table 1:** Summary of the related research.

Approach	Ref	Key Ideas
Distributed data communication constraints	[16]	Wireless communication in edge learning
	[17]	Deep neural network for fog-cloud based with adopting dynamic changes in resource variation
	[18]	Genetic algorithm for scheduling to minimize overall latency
ML at the Edge, Fog, and Cloud levels	[19]	Distributed ML and challenges for implementing (Hardware, security, privacy, and communication)
	[20]	A fruitful survey on distributed machine learning
	[21]	Proposed distributed gradient descent algorithm which fits for non-iid data
Federated ML concepts and applications	[9]	Stochastic method with variance reduction for solving the problem on federated learning
	[10]	Challenges of non-iid Data to Model Training on horizontal and vertical FL
	[11]	Overview of FL, technologies, protocols and applications
	[12]	Horizontal federated learning, vertical federated learning, and federated transfer learning
	[22]	Analyzing Fl regarding data partitioning, privacy, model, and communication
Federated optimization algorithms	[25]	FedAvg, FedProx, CO-OP, FSVRG
	[26]	FSVRG on fog or cloud
	[27]	FedProx
Distribution strategies and hierarchical FL	[28]	Hierarchical FL based on the number of aggregations compared to number of iterations (epochs)
	[29]	Hierarchical FL to minimize training loss and latency
Distributed ML for collaborative PM scenarios	[30]	Distributed PM algorithm based on FL and blockchain
	[13]	Cross-device FL for collaborative PM
	[31]	Real-time fault detection system for edge computing
	[32]	Edge computing in IoT based manufacturing
	[33]	Federated SVM for horizontal FL and federated random forest for vertical FL
	[34]	Novel FL algorithm for the LSTM model for anomaly detection
	[35]	Combination of CNN and LSTM in distributed anomaly detection applications

**Table 2:** Performance analysis of the synchronous FedSVM.

Optimizer	Evaluation Metrics	Dataset			
		FD001	FD002	FD003	FD004
GD	Runtime (s)	61.6	150	69	143.5
	Final acc (%)	92.4	77.9	94.2	78.4
SGD	Runtime (s)	18.9	43	20.5	45.5
	Final acc (%)	92.5	78.9	92.2	71.7
FSVRG	Runtime (s)	140	362	161	337
	Final acc (%)	90.3	74	91.7	86.8

**Table 3:** Performance analysis of the asynchronous FedSVM.

Optimizer	Evaluation Metrics	Dataset			
		FD001	FD002	FD003	FD004
GD	Runtime (s)	61.8	145	68.6	141.4
	Final acc (%)	92.2	77.2	93.8	77.1
SGD	Runtime (s)	19.8	42.5	21.4	43
	Final acc (%)	90	77.5	92.1	83.1

**Table 4:** Performance analysis of the synchronous FedLSTM.

Num of Epoch	Evaluation Metrics	Dataset			
		FD001	FD002	FD003	FD004
1	RMSE	13.33	22.83	12.57	25.1
	SF	242	226	156	560
2	RMSE	15.47	22.4	10.73	26.25
	SF	720	690	2469	470
3	RMSE	15.53	22.93	9.7	24.85
	SF	690	348	617	202
4	RMSE	14.5	21.68	9.65	17.14
	SF	709	753	895	337

**Table 5:** Performance analysis of the asynchronous FedLSTM.

Num of Epoch	Evaluation Metrics	Dataset			
		FD001	FD002	FD003	FD004
1	RMSE	16.14	22.11	14.68	29.5
	SF	174	2877	1452	492
2	RMSE	16.01	21.15	11.8	26.4
	SF	2097	2039	2769	3167
3	RMSE	15.81	21.16	11.87	26.1
	SF	410	1852	2796	2535
4	RMSE	15.36	22.29	11.85	27.1
	SF	1147	1473	5026	2650

**Table 6:** Results of other research for centralized RUL prediction on CMAPSS.

Prediction Model	Evaluation Metrics	Dataset			
		FD001	FD002	FD003	FD004
DCNN [38]	RMSE	12.61	22.36	12.64	23.31
	SF	273	10412	284	12466
Deep CNN [39]	RMSE	18.45	30.29	19.81	29.16
	SF	1286	13570	1596	7886
MODBNE [40]	RMSE	15.04	25.05	12.51	28.66
	SF	334	5585	6557	6557
CNN-XGB [41]	RMSE	12.61	19.61	13.01	19.41
	SF	224	2525	279	2930

**Table 7:** Performance analysis of FedSVM With MNIST dataset.

Optimizer	Evaluation Metrics	MNIST			
		Synchronous		Asynchronous	
		iid	Non-iid	iid	Non-iid
GD	Runtime (s)	109.84	97.15	93.89	86.26
	Final acc (%)	97.41	97.31	97.41	97.32
SGD	Runtime (s)	1.52	1.61	2.37	2.36
	Final acc (%)	97.69	97.32	96.86	96.76
SGD	Runtime (s)	328.93	332.68	-	-
	Final acc (%)	96.95	96.23	-	-

## Data Availability

The CMAPSS dataset is available in: https://data.nasa.gov/Aerospace/CMAPSS-Jet-Engine-Simulated-Data/ff5v-kuh6/ (accessed on 22 July 2022), and the MNIST dataset is available in: http://yann.lecun.com/exdb/mnist/ (accessed on 22 July 2022).

## Abbreviations

The following abbreviations are used in this manuscript:

PM	Predictive Maintenance
ML	Machine Learning
FL	Federated Learning
SVM	Support Vector Machine
LSTM	Long Short Term Memory
FedSVM	Federated Support Vector Machine
FedLSTM	Federated Long-Short Term Memory
RUL	Remaining Useful Life
DML	Distributed Machine Learning
Non-iid	Not Independent and Identically Distributed
CNNs	Convolutional Neural Networks
FSVRG	Federated Stochastic Variance Reduced Gradient
RNN	Recurrent Neural Network
RMSE	Root MEan Square Error
SF	Scoring Factor
